# Isolated central nervous system involvement in blastic plasmacytoid dendritic cell neoplasm: a rare case of relapse during systemic remission with diagnostic and therapeutic implications

**DOI:** 10.3389/fonc.2026.1818268

**Published:** 2026-04-15

**Authors:** Haocheng Zhao, Wenye Huang, Shanshan Lin, Yeping Chen

**Affiliations:** 1 Department of Hematology, Yueqing People’s Hospital, Yueqing, Zhejiang, China; 2 Department of Internal Medicine, Yueqing People’s Hospital, Yueqing, Zhejiang, China

**Keywords:** blastic plasmacytoid dendritic cell neoplasm (BPDCN), cerebrospinal fluid flow cytometry, intrathecal chemotherapy, isolated central nervous system relapse, systemic remission

## Abstract

Blastic plasmacytoid dendritic cell neoplasm (BPDCN) is a rare, highly aggressive hematologic malignancy characterized by frequent involvement of the skin and bone marrow. Central nervous system (CNS) infiltration is uncommon and may be clinically occult, thereby posing diagnostic challenges. We report the case of a 74 year-old man who initially presented with progressively enlarging cutaneous masses on the trunk. Histopathological examination of skin biopsy demonstrated BPDCN with a characteristic immunophenotype, and subsequent bone marrow studies confirmed extensive marrow involvement. The patient was treated with a combination of azacitidine and low-dose cytarabine to achieve sustained hematologic remission with marked regression of skin lesions and splenomegaly, despite intermittent treatment-related myelosuppression. After several months of stable disease, he was readmitted with new-onset paroxysmal occipital headache and blurred vision. Peripheral blood counts, bone marrow evaluation, and systemic imaging were unremarkable, and brain and orbital magnetic resonance imaging showed no abnormalities. Cerebrospinal fluid (CSF) analysis revealed marked pleocytosis with abundant atypical cells. Flow cytometry identified BPDCN cells accounting for 97.6% of nucleated cells, establishing the diagnosis of CNS involvement. Intrathecal methotrexate combined with cytarabine led to the rapid resolution of neurologic and visual symptoms. This case study demonstrates that BPDCN may lead to isolated CNS involvement despite ongoing systemic remission, highlighting the importance of cerebrospinal fluid evaluation in patients with unexplained neurologic manifestations.

## 1 Introduction

Blastic plasmacytoid dendritic cell neoplasm (BPDCN) is a rare, highly aggressive hematologic malignancy that arises from precursor plasmacytoid dendritic cells (1, 2). It mainly affects older adults and is characterized by a high propensity for dissemination and an unfavorable prognosis (1). Clinically, BPDCN most commonly presents with cutaneous lesions, frequently accompanied by bone marrow involvement and cytopenias, whereas lymph node and visceral infiltration may also occur (1, 2). Owing to its rarity and morphologic overlap with other hematologic neoplasms, the diagnosis of BPDCN is challenging and relies heavily on immunophenotypic confirmation, typically demonstrating expression of CD4, CD56, CD123, and other plasmacytoid dendritic cell–associated markers, in the absence of lineage-specific B- or T-cell antigens (2–5). Because the cutaneous manifestations of BPDCN are often heterogeneous, the disease may initially mimic other dermatologic or hematologic conditions, including eczema, lupus erythematosus, cellulitis, drug eruptions, cutaneous lymphoma, leukemia cutis, and cutaneous vasculitis. This clinical overlap may contribute to delayed diagnosis and underscores the importance of maintaining awareness of BPDCN in patients with atypical or unexplained skin lesions.

Central nervous system (CNS) involvement in BPDCN is uncommon and underrecognized in routine clinical practice (6). When present, it may manifest with nonspecific neurologic symptoms and can occur even in the absence of concurrent systemic progression (6, 7). Therefore, this poses a particular diagnostic challenge in patients who appear to be in hematologic remission, in whom neurologic complaints may be initially attributed to alternative etiologies (6). Conventional neuroimaging may be unremarkable, underscoring the importance of evaluation of the cerebrospinal fluid (CSF) (6, 7). CSF cytology and flow cytometry play a key role in identifying occult CNS infiltration and establishing a definitive diagnosis (6).

Here, we describe a rare case of BPDCN with CNS involvement that presented as an isolated relapse during sustained systemic remission (7). This report highlights the diagnostic value of CSF analysis and flow cytometry in such a setting and emphasizes the clinical relevance of considering CNS disease in patients with BPDCN who present with unexplained neurologic symptoms, with implications for timely diagnosis and targeted therapeutic intervention (6, 7).

## 2 Case presentation

**Figure 1:**
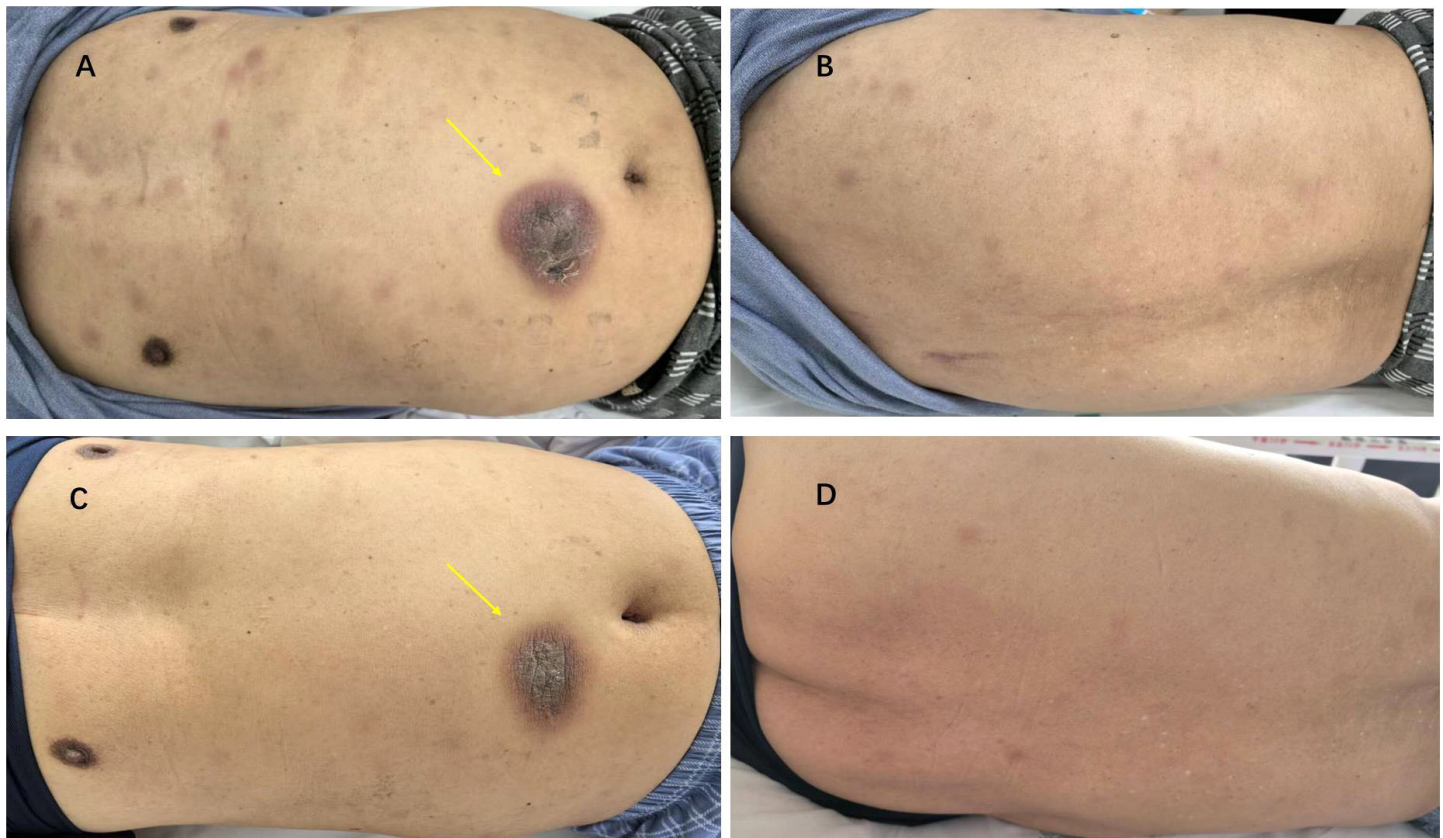
Clinical appearance of skin lesions before and after treatment. **(A)** Pre-treatment image showing a large, erythematous, violaceous plaque with a slightly ulcerated center on the right abdomen (yellow arrow), surrounded by multiple smaller macules and papules. **(B)** A closer view of the same area, demonstrating the extent of the skin lesions with evident erythema and nodular features. **(C)** Post-treatment image showing significant improvement, with the previously observed large plaque showing reduced size and a more flattened, less erythematous appearance (yellow arrow). The smaller lesions have also diminished in size and the extent of erythema. **(D)** Further lateral view after treatment, indicating resolution of the majority of lesions with minimal residual discoloration and no active inflammation.

**Figure 2:**
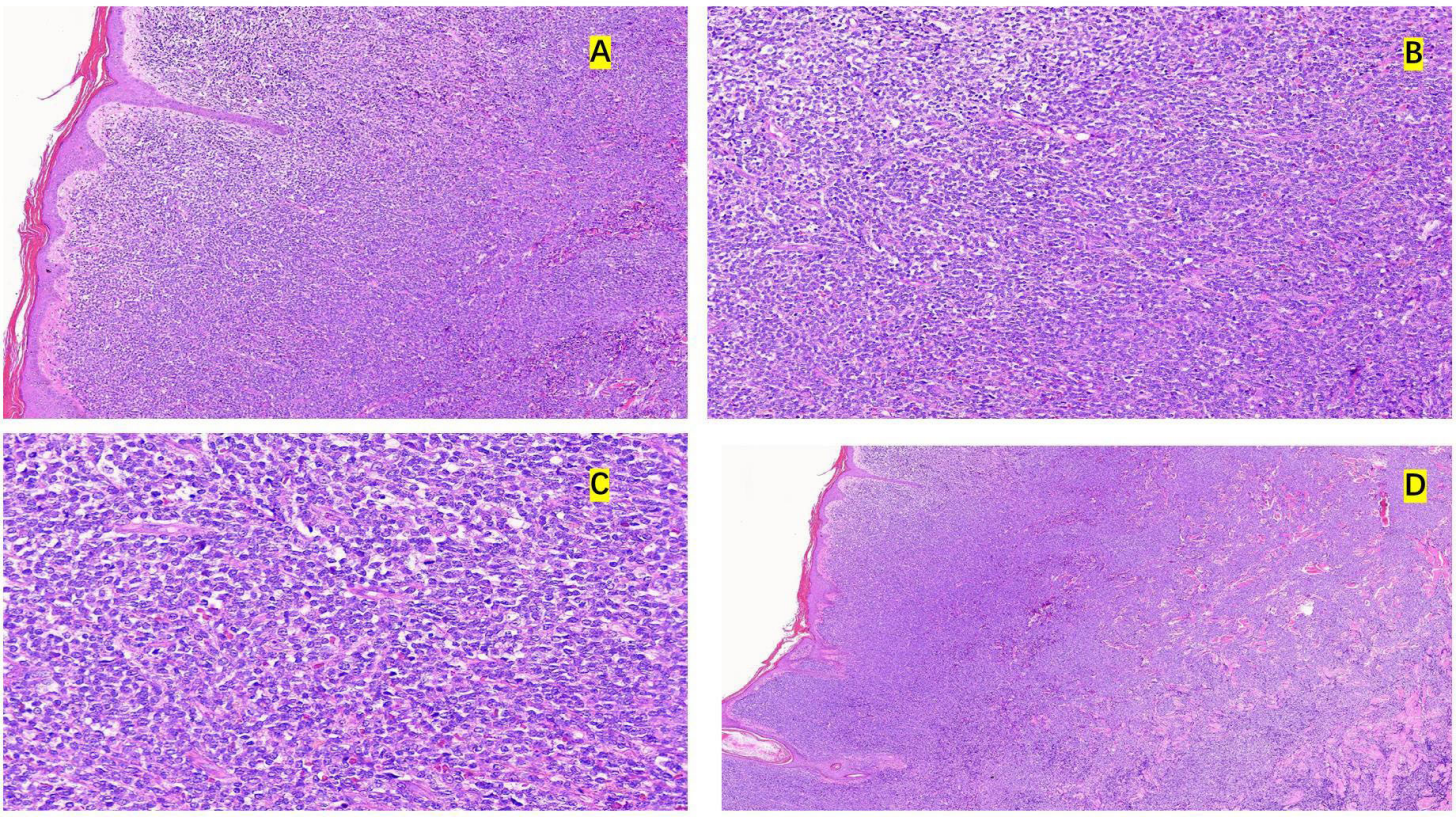
Histopathological findings from skin biopsy. **(A)** Low-magnification view showing dense diffuse infiltration of the dermis by neoplastic cells with relative sparing of the epidermis. **(B)** Intermediate-magnification view demonstrating a monomorphic population of medium-sized blastoid cells infiltrating the dermis. **(C)** High-magnification view showing tumor cells with irregular nuclei, fine to slightly coarse chromatin, scant cytoplasm, and focal plasmacytoid features. **(D)** Low-magnification view further illustrating the diffuse infiltrative growth pattern of neoplastic cells within the dermis. The diagnosis was further supported by immunohistochemistry, with tumor cells positive for CD43, CD56, BCL-2, and MUM1, a Ki-67 proliferation index of approximately 50%, and negative staining for CD4, CD34, and lineage-associated B- and T-cell markers.

A 74-year-old man without any relevant prior hematologic history presented with a 6-month history of progressively enlarging cutaneous lesions predominantly involving the trunk. Physical examination revealed multiple dense erythematous plaques and a prominent infiltrative violaceous plaque on the right abdomen, measuring approximately 7 cm × 6 cm, without ulceration or discharge (**Figure 1**). He denied any abdominal pain or constitutional symptoms. Initial laboratory findings demonstrated a white blood cell count of 4.23 × 10^^9^/L, hemoglobin of 112 g/L, thrombocytopenia (platelet count 44 × 10^^9^/L), and mildly elevated lactate dehydrogenase (LDH). Computed tomography (CT) revealed splenomegaly with heterogeneous density suggestive of splenic infarction or hemorrhage, as well as small axillary lymph nodes. A skin biopsy showed diffuse dermal infiltration by medium-sized blastoid cells. These cells were relatively monomorphic and showed irregular nuclei, scant cytoplasm, and a diffuse infiltrative growth pattern within the dermis. Immunohistochemistry demonstrated positivity for cluster of differentiation (CD)43, CD56, BCL-2, and Mum-1, and a Ki-67 index of approximately 50%, whereas lineage-specific B- and T-cell markers were negative, supporting a diagnosis of blastic plasmacytoid dendritic cell neoplasm (BPDCN) (**Figure 2**). Subsequent bone marrow examination revealed marked hypercellularity with approximately 65% of blastoid cells. On smear review, these cells were medium sized, with round to irregular nuclei, coarse chromatin, variable amounts of gray-blue cytoplasm, occasional nuclear folding, and focal plasmacytoid features. Bone marrow biopsy further showed diffuse infiltration by medium-sized blasts accounting for more than 80% of cells, with round nuclei, fine chromatin, occasional nucleoli, and relatively abundant cytoplasm. Bone marrow flow cytometry revealed BPDCN cells accounting for 69.4% of nucleated cells, expressing CD123, CD4, and CD56, with negative CD303 and dim CD304 expression; bone marrow biopsy confirmed extensive involvement with CD123 and CD56 expression, establishing the diagnosis. Although CD4 expression was absent in the skin biopsy, the overall immunophenotypic profile, together with bone marrow and CSF flow cytometry findings, supported the diagnosis of BPDCN. A clinical timeline summarizing the major events of the case is provided in **Table 1**.

**Table 1:** Clinical timeline of the patient’s disease course, diagnostic evaluation, treatment, and central nervous system relapse.

Time point	Clinical event	Key findings / management
At presentation	Progressive cutaneous lesions on trunk	Multiple erythematous plaques and a prominent violaceous abdominal plaque
Initial workup	Laboratory and imaging evaluation	Thrombocytopenia, mildly elevated lactate dehydrogenase, splenomegaly on CT
Diagnosis	Skin biopsy and bone marrow evaluation	Skin biopsy and bone marrow studies consistent with BPDCN
Initial treatment	Azacitidine + low-dose cytarabine	Lower-intensity treatment initiated
During follow-up	Systemic remission	Regression of skin lesions, reduction in splenomegaly, sustained hematologic remission
~9 months later	New neurologic symptoms	Paroxysmal occipital headache and blurred vision
Relapse evaluation	CNS assessment	Brain/orbital MRI unremarkable; CSF cytology and flow cytometry confirmed CNS involvement
CNS-directed treatment	Intrathecal methotrexate + cytarabine	Rapid improvement in headache and visual symptoms
Ongoing management	Follow-up	Planned additional intrathecal therapy and continued systemic treatment

The patient was treated with a combination of azacitidine (100 mg, days 1–7) and low-dose cytarabine (50 mg, days 1–5). Treatment was complicated by episodes of severe myelosuppression, which were managed with supportive care, following which hematologic recovery was achieved. Serial imaging and clinical evaluations demonstrated a marked regression of cutaneous lesions, reduction in splenomegaly, and sustained hematologic remission. The patient subsequently received multiple cycles of the same regimen, which maintained ongoing disease control.

**Figure 3:**
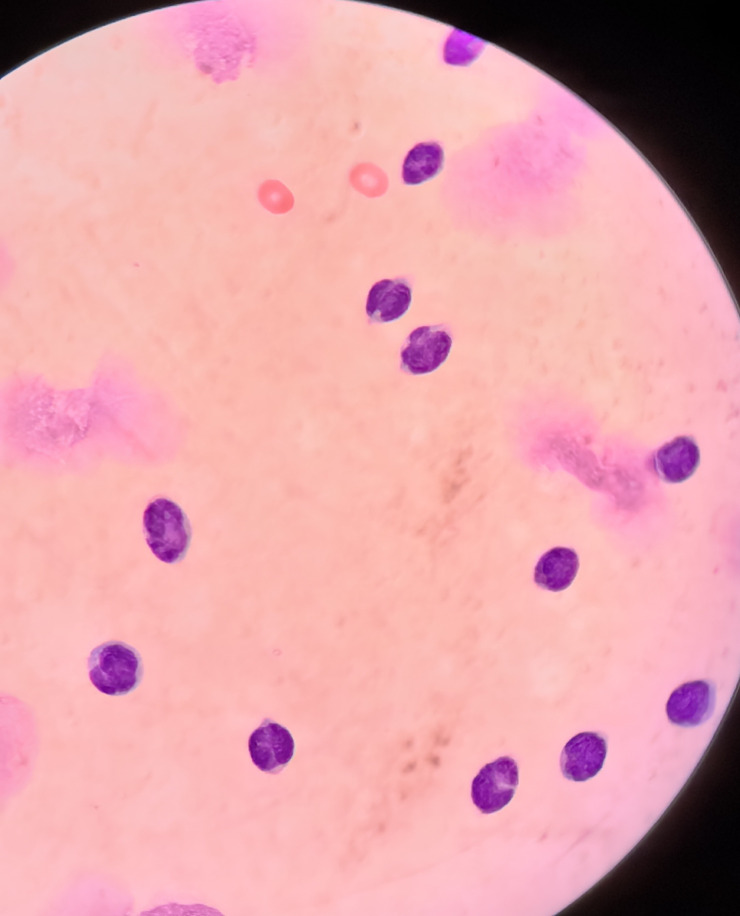
Cerebrospinal fluid (CSF) conventional cytology with Wright’s stain (oil immersion view). Image showing a CSF sample from the patient with BPDCN, highlighting atypical blast-like cells with a high nuclear-to-cytoplasmic ratio, round nuclei, dispersed chromatin, and a prominent nucleolus. The scant basophilic cytoplasm is typical of malignant plasmacytoid dendritic cells.

**Figure 4:**
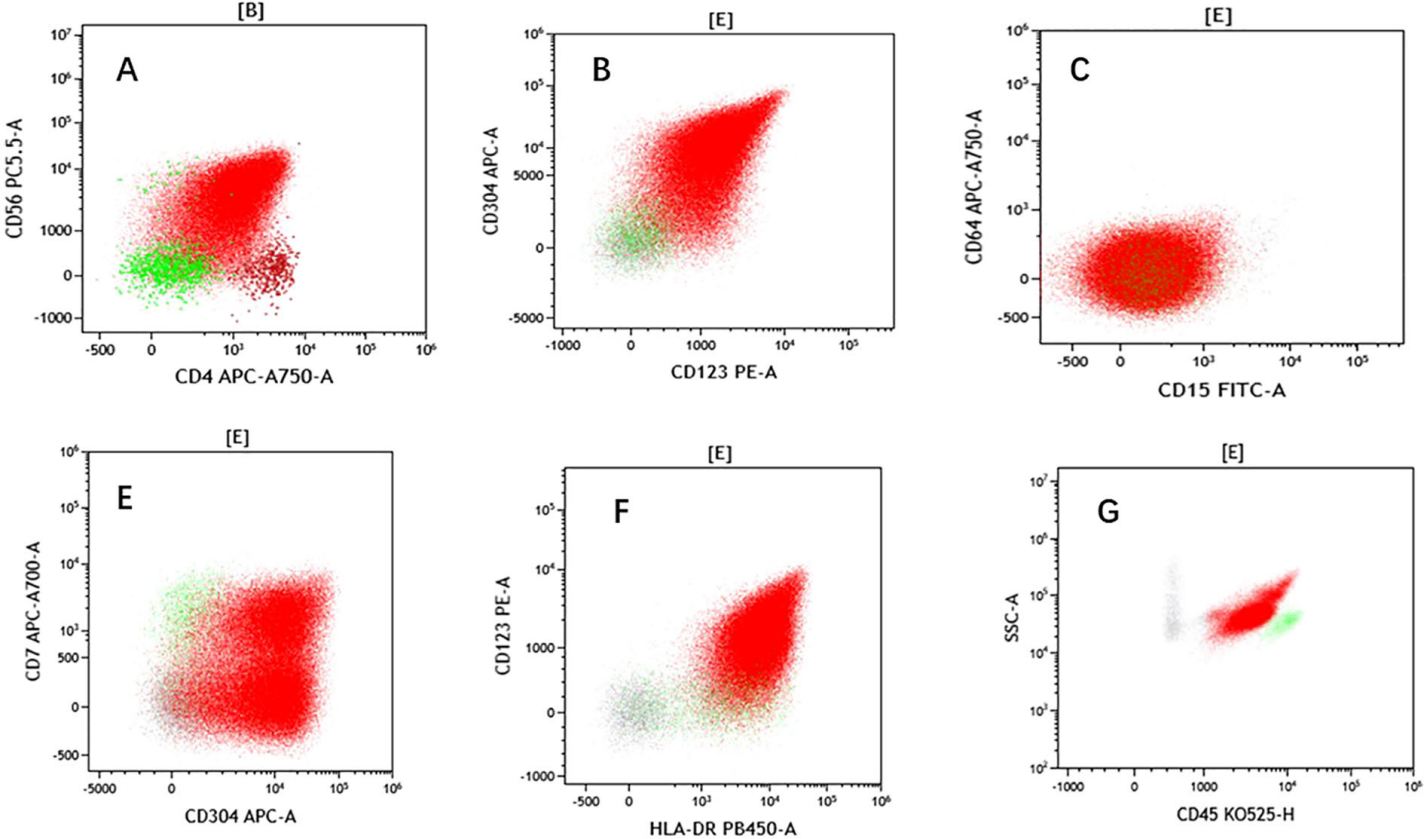
Cerebrospinal fluid (CSF) flow cytometry. **(A)** Before treatment: Flow cytometry showing a population of cells expressing CD56 and CD4, with a clear separation of these markers into distinct clusters. **(B)** A distinct CD304^+^ population is observed, consistent with plasmacytoid dendritic cells. **(C)** CD64 expression is absent in the abnormal cell population, supporting exclusion of monocytic leukemia in the differential diagnosis. **(D)** CD7 is partially expressed in the abnormal cell population, which is a recognized immunophenotypic finding in BPDCN. **(E)** HLA-DR expression is detected in a portion of the cells, aligning with BPDCN-associated characteristics. **(F)** Strong CD123^+^ expression is evident, highlighting the relevant cell population. **(G)** A scatter plot of CD45 versus side scatter indicates a distinct population with altered light-scattering properties, characteristic of BPDCN cells in the CSF.

Approximately 9 months after achieving stable remission, the patient developed new-onset paroxysmal occipital headache accompanied by bilateral blurred vision. Peripheral blood counts remained stable. Repeat bone marrow evaluation and systemic imaging showed no evidence of disease progression. Brain and orbital magnetic resonance imaging were unremarkable. Lumbar puncture was performed, given the persistent neurologic symptoms of the patient. CSF analysis revealed marked pleocytosis with a predominance of lymphoid cells and numerous atypical mononuclear cells on cytologic examination (**Figure 3**, **Table 2**). Flow cytometry of the CSF demonstrated BPDCN cells comprising 97.6% of nucleated cells, with the expression of CD123, CD4, CD56, CD304, human leukocyte antigen–DR, CD38, and CD33, confirming CNS involvement (**Figure 4**).

**Table 2:** Cerebrospinal fluid routine and biochemical analysis.

Category	Test item	Result	Unit	Reference range
Routine	Color	Pale yellow	—	—
Routine	Clarity	Slightly cloudy	—	—
Routine	Nucleated Cell Count	3159 ↑	/µL	0–8 /µL
Routine	Red Blood Cell Count	600 ↑	/µL	<1 /µL
Routine	Pandy’s Test	Positive ↑	—	Negative
Routine	Neutrophils	1	%	0–6 %
Routine	Lymphocytes	91 ↑	%	40–80 %
Routine	Macrophages	8	%	—
Biochemical	Glucose Test	3.00	mmol/L	2.24–4.48 mmol/L
Biochemical	Chloride	118.5 ↓	mmol/L	120–130 mmol/L

In the routine CSF differential count, the abnormal tumor cells were morphologically categorized within the lymphocyte population on Wright-stained cytologic examination; their neoplastic nature was subsequently confirmed as BPDCN cells by CSF flow cytometry.

The patient was promptly treated with the combination of intrathecal methotrexate (10 mg) and cytarabine (50 mg). Neurologic symptoms, including headache and visual disturbance, improved rapidly within a day of treatment. He is now being actively managed with planned additional intrathecal therapy and continued systemic treatment, with close clinical and laboratory follow-up.

## 3 Discussion

BPDCN is a rare and highly aggressive hematologic malignancy derived from precursor plasmacytoid dendritic cells, predominantly affecting older adults (1, 2). This condition is characterized by early dissemination and an unfavorable prognosis (1). Cutaneous involvement is the most common initial manifestation, and it frequently precedes or accompanies bone marrow infiltration and cytopenias (1, 2). However, the clinical and morphologic heterogeneity of BPDCN, together with its overlap with acute leukemias and other hematologic malignancies, often leads to diagnostic delays (1, 3, 4, 8). Thus, accurate diagnosis relies on integrated histopathologic and immunophenotypic evaluation. Classically, BPDCN cells express plasmacytoid dendritic cell-associated markers, including CD123, CD4, CD56, TCL1, and CD303/CD304, while lacking lineage-specific B- or T-cell antigens (2, 5). Although the histologic and immunohistochemical findings in the skin biopsy were suggestive of BPDCN, they were not fully definitive in isolation. In this case, the final diagnosis was established through integrated clinicopathologic and immunophenotypic evaluation, including bone marrow examination and flow cytometric findings from both bone marrow and cerebrospinal fluid. Despite advances in therapy, including hypomethylating agents and CD123-targeted treatments, long-term disease control remains challenging, particularly in older patients who are not candidates for intensive chemotherapy or allogeneic hematopoietic stem cell transplantation (1, 2, 9–12). Although tagraxofusp is an approved CD123-targeted therapy for BPDCN, it was not available at our institution or at larger regional referral centers during this patient’s treatment course. In addition, the patient’s family declined pursuing further external arrangements to obtain this agent. Therefore, given the patient’s advanced age and the need for a practical lower-intensity approach, azacitidine combined with low-dose cytarabine was selected as a feasible treatment option in this real-world setting.

The CNS represents a clinically important but underrecognized disease compartment in BPDCN (6). Although considered rare historically, emerging evidence suggests that CNS infiltration may occur more frequently than previously reported, including cases of occult or isolated involvement (6, 7). Clinically, CNS disease may occur at diagnosis, during treatment, or at relapse and can often present with nonspecific neurologic symptoms such as headache, visual disturbance, or cranial neuropathies (6, 7). Difficulties in diagnosing are further compounded in patients who appear to be in systemic remission, as neurologic symptoms may be attributed to treatment-related toxicity, vascular events, infection, or age-related neurologic conditions (6). Importantly, conventional neuroimaging may appear normal despite substantial leptomeningeal disease, increasing the risk of delayed or missed diagnosis (6, 7).

The present case exemplifies the listed challenges. The patient initially presented with prominent cutaneous disease and bone marrow involvement and achieved sustained systemic remission with the combination of azacitidine and low-dose cytarabine (2, 13). Months into disease control, the patient developed new-onset headache and visual disturbance, while peripheral blood counts, bone marrow evaluation, and systemic imaging showed no evidence of progression (6, 7). Brain and orbital magnetic resonance imaging were unremarkable (6, 7). Ultimately, CSF analysis established the diagnosis of CNS involvement, revealing marked pleocytosis with atypical cells on cytology and a dominant BPDCN population as determined using flow cytometry (6, 7). This clinical trajectory underscores a key message that is being increasingly emphasized in the literature: normal imaging and apparent systemic remission do not exclude CNS disease in BPDCN, and CSF evaluation is indispensable when neurologic symptoms are unexplained (6, 7).

From a diagnostic perspective, this case study highlights the complementary roles and limitations of the available modalities. Neuroimaging is essential in excluding parenchymal lesions or alternative etiologies, but it lacks sensitivity for isolated leptomeningeal disease (6, 7). Conventional CSF analysis and cytology can suggest malignant involvement, but these modalities are limited by low sensitivity and morphologic ambiguity (6). In contrast, CSF flow cytometry is a highly sensitive technique that can be used for objective immunophenotypic confirmation, particularly when the CSF phenotype can be directly correlated with known systemic disease (6). In this patient, flow cytometry was used to identify BPDCN cells comprising 97.6% of nucleated CSF cells, conclusively establishing the diagnosis and resolving diagnostic uncertainty (6). Notably, CD4 was absent in the initial skin biopsy but was detected in later bone marrow and CSF specimens, suggesting immunophenotypic heterogeneity across disease compartments; technical variability in the skin specimen cannot be completely excluded. These findings support the early incorporation of CSF flow cytometry into the diagnostic algorithm for patients with BPDCN who develop neurologic symptoms, even in the absence of radiologic abnormalities (6, 7).

Therapeutic strategies for BPDCN with CNS involvement are not standardized and are largely extrapolated from treatment paradigms for acute leukemia and aggressive lymphomas (2, 6). CNS-directed therapy, most commonly intrathecal chemotherapy with methotrexate and/or cytarabine, is the cornerstone of management (6, 7). In the present case, CNS prophylaxis was not administered at initial diagnosis because there were no neurologic symptoms or other clinical findings suggestive of CNS involvement at that time. Although it cannot be determined retrospectively whether earlier CNS-directed therapy would have altered the subsequent disease course, this case highlights the possibility that occult CNS disease may already be present in BPDCN despite an apparently controlled systemic disease status. Accordingly, earlier CSF evaluation and consideration of CNS-directed strategies may merit discussion in selected patients. In our patient, the combination of intrathecal methotrexate and cytarabine led to a rapid and marked improvement in neurologic and visual symptoms, highlighting the efficacy of prompt CNS-directed intervention (6, 7). Nevertheless, durable disease control likely requires ongoing CNS surveillance integrated with systemic therapy, as isolated CNS involvement may precede systemic relapse or coexist with occult disease reservoirs (6, 7). The optimal systemic approach, particularly in patients who are older or frail, is undefined, warranting further investigation (2, 10).

The novelty of this case study lies in the presentation of isolated CNS involvement emerging during sustained systemic remission, with negative neuroimaging and diagnosis established exclusively on the basis of CSF cytology and high-sensitivity flow cytometry (7). This pattern has important clinical implications, indicating that apparent systemic disease control does not exclude the possibility of CNS involvement (6, 7). Clinicians should therefore maintain a high index of suspicion for CNS involvement in patients with BPDCN who present with unexplained neurologic symptoms, regardless of their systemic disease status (6, 7). Early CSF evaluation and the timely initiation of CNS-directed therapy may help prevent irreversible neurologic injury and improve clinical outcomes (6, 7).

Our study has some limitations. This report describes a single patient, which limits generalizability and precludes conclusions related to the incidence, optimal screening strategies, or comparative efficacy of CNS-directed therapies (6). In addition, long-term outcomes such as durability of CNS response and subsequent systemic disease course are not yet available (6). Molecular and cytogenetic data were not included; including these could provide insights into the biological mechanisms underlying CNS tropism in BPDCN (5, 14). Future studies should therefore focus on defining the true prevalence and timing of CNS involvement, identifying clinical and biologic predictors of CNS disease, and optimizing integrated CNS and systemic treatment strategies based on prospective investigation (6, 15).

## 4 Conclusions

This case study underscores the rare, clinically significant occurrence of isolated CNS involvement in BPDCN, a highly aggressive hematologic malignancy characterized by cutaneous and bone marrow involvement. Despite systemic remission that was achieved using the combination of azacitidine and low-dose cytarabine, the patient developed new-onset neurologic symptoms, including headache and blurred vision, months after initial treatment. The diagnostic challenge in this case arose from the absence of abnormal neuroimaging, highlighting the need for heightened suspicion of CNS relapse even when systemic disease appears controlled. CSF analysis and flow cytometry were used to confirm CNS involvement, underscoring the role of these diagnostic techniques in detecting the relapse of BPDCN in the CNS, particularly in patients with negative imaging.

The clinical implications of this case are profound and emphasize the necessity of vigilant CNS monitoring in patients with BPDCN, even during phases of systemic remission. It reinforces the diagnostic importance of CSF analysis and flow cytometry for detecting occult CNS disease that may be missed during conventional imaging. Prompt intrathecal chemotherapy resulted in the rapid resolution of symptoms in the patient, highlighting the importance of early, targeted CNS therapy.

Findings from this case study suggest the need for future research to better understand the mechanisms underlying CNS involvement in BPDCN, refine early diagnostic methods, and optimize treatment strategies for CNS relapse. Prospective studies are warranted to evaluate the long-term efficacy of CNS-directed therapies, particularly for use in combination with systemic treatments.

Certain limitations of this case study should be acknowledged, including the single-patient nature of the report, which limits the generalizability of our findings and the possibility to assess broader clinical implications. Future studies involving larger cohorts are therefore necessary to confirm CNS involvement in BPDCN, establish standardized diagnostic protocols, and develop evidence-based treatment strategies for early detection and intervention for CNS relapse.

## Data Availability

The original contributions presented in the study are included in the article/supplementary material. Further inquiries can be directed to the corresponding author.

## Glossary

BPDCN: blastic plasmacytoid dendritic cell neoplasm
CNS: central nervous system
CSF: cerebrospinal fluid
CT: computed tomography
MRI: magnetic resonance imaging
HLA-DR: human leukocyte antigen–DR
